# Hand-Crafted and Learned Feature Aggregation for Visual Marble Tiles Screening

**DOI:** 10.3390/jimaging8070191

**Published:** 2022-07-08

**Authors:** George K. Sidiropoulos, Athanasios G. Ouzounis, George A. Papakostas, Anastasia Lampoglou, Ilias T. Sarafis, Andreas Stamkos, George Solakis

**Affiliations:** 1 MLV Research Group, Department of Computer Science, International Hellenic University, 65404 Kavala, Greece; georsidi@cs.ihu.gr (G.K.S.); athouzo@emt.ihu.gr (A.G.O.); anlabog@mst.ihu.gr (A.L.); 2 Department of Chemistry, International Hellenic University, 65404 Kavala, Greece; isarafis@chem.ihu.gr; 3 Intermek A.B.E.E., 64100 Kavala, Greece; a.stamkos@intermek.gr; 4 Solakis Antonios Marble S.A., 66100 Drama, Greece; george@solakismarble.gr

**Keywords:** marble tile sorting, deep learning, machine learning, texture description, CNN, feature fusion

## Abstract

An important factor in the successful marketing of natural ornamental rocks is providing sets of tiles with matching textures. The market price of the tiles is based on the aesthetics of the different quality classes and can change according to the varying needs of the market. The classification of the marble tiles is mainly performed manually by experienced workers. This can lead to misclassifications due to the subjectiveness of such a procedure, causing subsequent problems with the marketing of the product. In this paper, 24 hand-crafted texture descriptors and 20 Convolution Neural Networks were evaluated towards creating aggregated descriptors resulting from the combination of one hand-crafted and one Convolutional Neural Network at a time. A marble tile dataset designed for this study was used for the evaluation process, which was also released publicly to further enable the research for similar studies (both on texture and dolomitic ornamental marble tile analysis). This was done to automate the classification of the marble tiles. The best performing feature descriptors were aggregated together in order to achieve an objective classification. The resulting model was embodied into an automatic screening machine designed and constructed as a part of this study. The experiments showed that the aggregation of the VGG16 and SILTP provided the best results, with an AUC score of 0.9944.

## 1. Introduction

**Figure 1:**
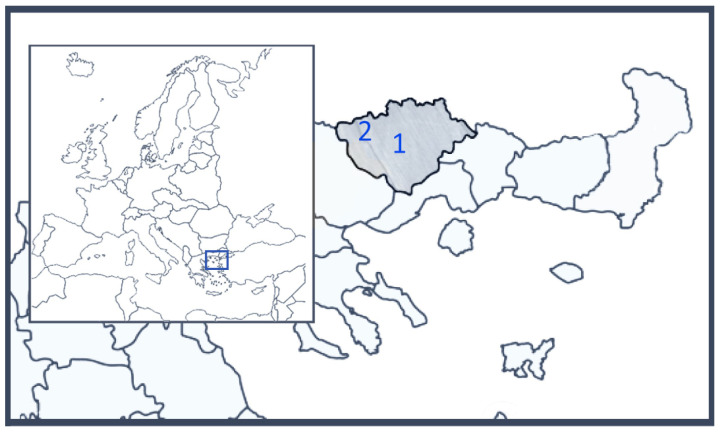
The location of (1) the city of Drama, (2) the dolomitic marble quarry at the village of Kokinoghia in the regional unit of Drama.

The marble industry holds an important share in the economic life of the regional unit of Drama, which belongs to the administrative region of Eastern Macedonia and Thrace in Northern Greece (Figure 1). In the Falakron mountain area, many excellent varieties of dolomitic marble [1] are quarried and shipped all over the world. Dolomitic marbles are a magnesium (Mg)-rich variant of marble and are generally of better quality than calcite (Ca)-rich marble. One of the best products, Grey Lais, is quarried by Solakis S.E.

The key factor to success, besides the high quality of the raw material quarried, is the classification of the different types of tiles into different categories based on the ornamentation. This is still performed manually and, therefore, can, in some cases, lead to the shipment of tiles that do not correspond to the same quality level. Texture analysis is a valuable tool in many real-world applications, and it is also a very promising technology to automate the quality classification of the natural stone dolomitic marble tiles and thus, boost the price of the product.

Since 2005 many scientific papers have been published about the classification of ornamental natural stones using machine learning (ML) techniques. Most research has been performed on marble. In 2005 an attempt was made to classify “Crema Marfil Sierra de la Puerta” marble slabs into three categories. The achieved classification rate was 98.9% [2]. Using mathematical morphology segmentation and classification of colored and polished marble tiles was achieved [3]. Convolutional Neural Network (CNN) approaches were first applied to granite tile classification in 2017. In this approach, small patches of images taken from granites were used in order to augment the dataset, and a majority voting procedure was taken into account [4]. In 2010, functional neural networks were tested in order to classify granite tiles [5]. In 2019, DNNs were also used for the first time with promising results [6]. In 2020, the VISUAL Geometry Group 16 (VGG16) CNN was used to identify images of peridotite, basalt, marble, gneiss, conglomerate, limestone, granite, and magnetite quartzite with a recognition probability greater than 96% [7].

Our research team has been working since 2020 to develop an automatic pipeline to replace the manual classification of natural stone tiles made from marble slabs. In 2021, three papers were published by our team regarding this topic. In the first paper, 24 image descriptors were tested with 7 classifiers [8]. The results showed that the Extreme Gradient Boost (XGB) classification algorithm [9] performed best with the XCS-LBP [10] texture descriptor. In the second paper [11], 15 Convolutional Neural Networks (CNN) were examined. The results showed that the DenseNet201 [12] performed best in this task. Furthermore, the results were interpreted using Gradient-weighted Class Activation Mapping (Grad-CAM) [13]. In the third paper, regression rather than classification was used to assign quality values to the marble tiles. In this case, MobileNetV2 (MNV2) [14] achieved the best results [11]. The next step of this research was to investigate if the aggregation of hand-crafted descriptors (HCDs) and CNNs could further improve the performance of the dolomitic marble tile classification.

The contribution of this paper can be summarized in the following four points. First, to highlight the improvement regarding the classification accuracy, 20 HCDs and 24 CNNs are first presented and evaluated in terms of their ability to classify dolomitic marble tiles into predefined quality categories. Secondly, the aggregation of HCDs and CNN-based types of features for the classification of marble tiles is presented for the first time in the literature. As a result, the aforementioned aggregation leads to the classification of tiles with very high accuracy, solving the issue of this categorization in realistic conditions (as part of a larger, integrated production system). Lastly, the release of such a dataset will further enable the research on texture analysis in real-life scenarios, as well as the analysis of dolomitic ornamental marble tiles regarding their quality or other features.

Due to the nature of the problem that this study aims to solve, that is, texture classification, the HCDs that are employed have been extensively used in the literature to solve such problems. In addition to that, due to the deep architecture and a large number of layers of CNNs, they have the ability to perform their own type of (automatic) feature extraction. On the one hand, HCDs have the ability to extract local features from the images, generating features that can be visually understood, while CNNs extract global and abstract features from the images. Therefore, the aggregation of those two types of features allows us to solve this classification problem, in the context of a production system, with a very high accuracy rating. In this study, first, the performance of each HCD and CNN model is evaluated separately so as to present their performance and, as a result, highlight the important improvement regarding the results when they are aggregated to a single feature vector.

In Section 2, the characteristics of the marble texture are discussed. Section 3 presents an in-depth analysis of the proposed methodology. The results are presented in Section 4. This paper closes with a discussion and the conclusion in Section 5 and Section 6, respectively.

## 2. Dolomitic Marble Texture Description

Polished tiles of dolomitic marble from the quarries in the Kokkinoghia region present a non-stationary texture. Furthermore, no two tiles are 100% identical. The ornamentation is the result of the metamorphism and deformation of initially layered sedimentary rocks, which make up 94% of the light-colored mineral dolomite $CaMg{\left(CO_{3}\right)}_{2}$ and 6% of the darker $CaCO_{3}$ calcite [15].

**Figure 2:**
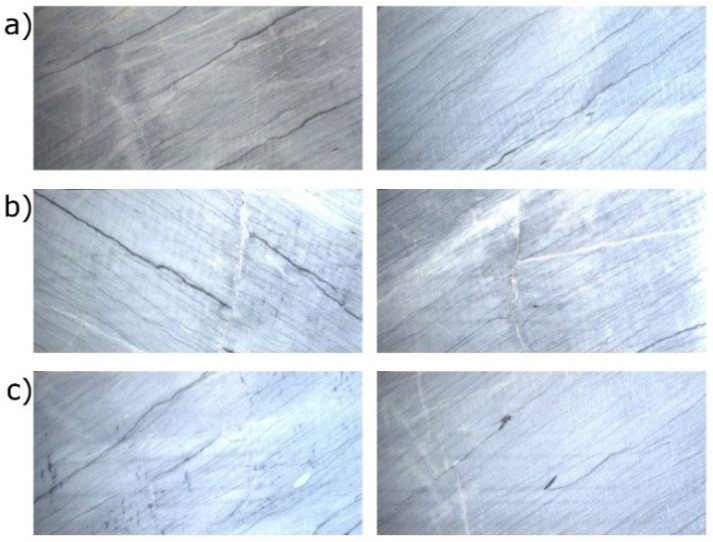
The three types of marble tiles used in the dataset supplied by Solakis S.A. (**a**) Class A, (**b**) class B, and (**c**) class C.

Due to the wide range of ornamentation encountered in the polished tiles, it is possible to establish a wide range of classes. Nevertheless, the current marketing strategies of Solakis S.A. imposed a three-fold classification. These three classes can be described as follows. Class A has a fine bedded parallel ornamentation consisting of dark (calcite) and light-colored (dolomite) lines (Figure 2a). In Class B, tile cracks of random angles are present and cut the motive of Class A (Figure 2b). These cracks are the result of post-metamorphic tectonization. In Class C, patches of dark-colored calcite create unwanted impurities (Figure 2c).

## 3. Methodology

### 3.1. Dataset Description

The initial dataset, exclusively created for this research project, consists of 982 stone tiles, with an original size of 30 × 60 cm. The dataset, which was manually classified into three classes by specially trained workers, was extremely unbalanced. To overcome this problem, the dataset was reduced to 441 tiles, with each class consisting of 147 samples. The dataset is available on GitHub (https://github.com/MachineLearningVisionRG/d-dom-dataset) (accessed on 6 July 2022).

### 3.2. Dataset Acquisition

**Figure 3:**
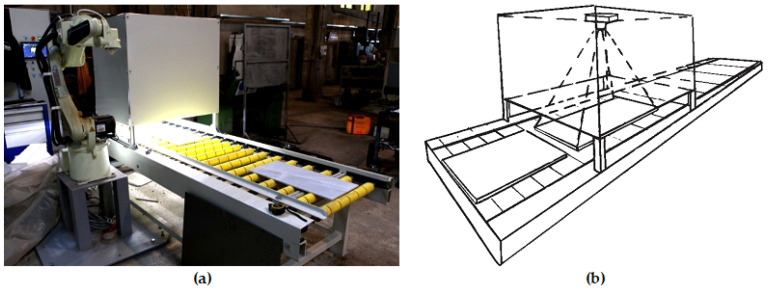
Image acquisition. (**a**) The automatic screening machine constructed by Intermek A.B.E.E., (**b**) schematic of the digital image acquisition inside the diffuser box.

The tiles were fed manually onto a mechanical roller table, which moved the tile into the diffusion box, where the digital image was acquired on the fly. At the exit, a mechanical arm transports the tile onto the corresponding class’s pile. This automatic screening machine designed and constructed my INTERMEK A.B.E.E. (Figure 3a) consists of the diffusion box where the digital image is captured and the robotic arm that is moving the marble tiles to one of three piles based on the developed ML model.

The marble tile digital images were acquired by a MV_CA050-10GM/GC digital camera equipped with a MVLMF0824M-5MP lens at a 90 cm distance. L.E.D. arrays were used as a light source inside a diffusion box (Figure 3b).

### 3.3. Hand-Crafted Descriptor Learning

In our experiments, 24 texture descriptors are included (Table 1): two key-point detectors and descriptors, namely Oriented FAST and rotated BRIEF (ORB) [16] and Scale Invariant Feature Transform (SIFT) [17]. A total of 17 local pattern descriptors that are divided into four categories: Four local ternary patterns (LTP), namely: SILTP [18], CSLTP [19], CSSILTP, and XCSLTP [20]; Two local derivative patterns (LDP), namely: Center-Symmetric LDP (CSLDP) [21] and Center-Symmetric Local Deritative Mapped Pattern (CSLDMP). Two local mapped patterns (LMP), namely: eXtended Center-Symmetric LMP (XCSLMP) [22] and Center-Symmetric LMP (CSLMP) [23]. Nine local binary patterns LBP), namely: eXtended Center-Symmetrical LBP (XCSLBP) [10], Center-Symmetric LBP (CSLBP) [24], Elliptical-LBP (ELBP) [25], LBP-NRI Uniform [26], LBP-ROR [27], LBP-Uniform [28], OLBP [29], SCSLBP [30], and VARLBP [28]. Five other types of descriptors: Haralick [31], Gabor [32], GLCM [33], Histogram of Oriented Gradients (HOG) [34], and TAS [35].

For the extraction of the aforementioned descriptors, the LBP Library [10], Local Descriptors for Image Classification [36], Mahotas [37], and Scikit-image libraries were used. Moreover, the LBP-NRI Uniform corresponds to the non-rotation-invariant uniform patterns variant of the LBP descriptor; the LBP-ROR to rotation invariant and LBP-Uniform to an improved rotation and grayscale invariant version of the descriptor.

The reasoning behind the choice of the aforementioned descriptors is due to their wide use in texture classification problems and extensive application for texture feature extraction in various cases, from biometric identification [38] and character recognition [39] to texture classification [40,41,42] and others [43,44].

**Figure 4:**
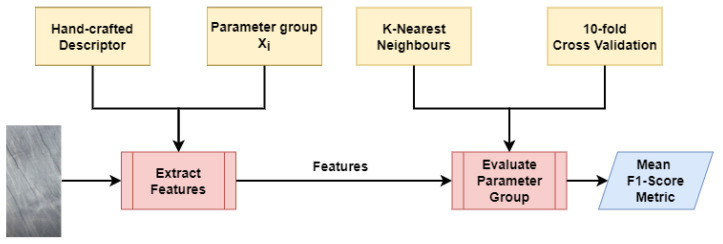
Flowchart of the evaluation process for a single parameter group of an HCD.

As the feature vector of each descriptor depends heavily on the parameters chosen, a grid search hyperparameter optimization algorithm was employed. The performance of each group of parameters was evaluated according to the F1-score of a K-Nearest Neighbors classifier. This process is better illustrated in Figure 4.

It should be noted that, in the cases of HOG, ORB, and SIFT, as the feature vector that was being extracted was very large, PCA was applied, with the number of components explaining at least 90% of the variance. For the LTP, LDP, LMP, and LBP types of features, a density histogram with varying bins was computed and used as the feature vector. As for the Gabor feature, the feature extraction process included the following steps:

1. calculate the real and imaginary response of the filter applied to the image,
2. calculate the magnitude between the real and imaginary response, and
3. calculate the mean and standard deviation of the magnitudes from all the filters.

Specifically, a total of 12 filters were being applied to each image, derived from the combination of 3 frequencies (i∗sqrt(i), with iϵ{2,6,10}) and 4 orientations (3 linearly spaced values ϵ[0,π]).

After finding the best parameters for each descriptor, 7 estimators were employed and evaluated, and their results were compared, namely:

1. Support Vector Machine (SVM) (with RBF kernel),
2. K-Nearest Neighbor (kNN),
3. Random Forest (RF),
4. Multilayer Perceptron (MLP),
5. Logistic Regression (LR),
6. Stochastic Gradient Descent (SGD),
7. Extreme Gradient Boost (XGB).

**Figure 5:**
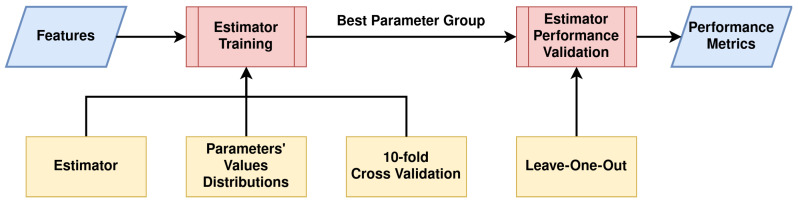
Flowchart of the training and validation process of an estimator.

Similarly, a hyperparameter optimization was applied for each estimator, using the Bayesian search algorithm provided by the Scikit-optimize library. This technique tests a fixed number of parameter settings from specified distributions, contrary to the exhaustive grid search that runs through all the parameter combinations. The performance of each group of parameters was evaluated using the mean F1-score over a 10-fold cross-validation technique. The training process of a single estimator for a given HCD is depicted in Figure 5.

### 3.4. Convolutional Neural Network Training

It is obvious that the available dataset is very small to train a CNN from scratch. Therefore, a transfer-learning technique was employed on 20 state-of-the-art CNNs, namely (Table 2): DenseNet121 (DN121), DenseNet169 (DN169), DenseNet201 (DN201) [45], EfficientNetB0 (ENB0), EfficientNetB4 (ENB4), EfficientNetB6 (ENB6) [46], InceptionResNetV2 IRNV2, InceptionV3 (IV3) [47], MobileNet (MN), MobileNetV2 (MNV2) [14], NASNetMobile (NASNM) [48], ResNet101 (RN101), ResNet101V2 (RN101V2), ResNet152 (RN152), ResNet152V2 (RN152V2), ResNet50 (RN50), ResNet50V2 (RN50V2) [49], VGG16, VGG19 [7], and Xception (XC) [50].

Those CNNs were pretrained on the ImageNet database and are available from the Keras Library. The reasoning behind choosing those specific networks and applying transfer learning techniques, in general, is manifold. First, the application of conventional ML models (such as the ones mentioned in Section 3.3) has the important limitation of requiring a lot of data that have the same distribution between classes. For this reason, the study employs homogeneous transfer learning (same feature space), which transfers the knowledge across domains [51], so as to solve the present problem. To perform the transfer learning process, models that have already been trained in other domains are required, and, therefore, the aforementioned available models were employed. Secondly, those models are considered state-of-the-art models, having very good results when tested on the ImageNet database (above 70% Top-1 Accuracy). Additionally, these models have been applied in numerous studies to tackle other problems through transfer learning techniques [52,53,54], performing satisfyingly without the need to design and build models from scratch. Moreover, a very important limitation in many studies regarding the choice of model is the size of the network and inference speed. Many applications require the use of lightweight models or models that can predict the target output fast. However, those limitations do not apply in this study, and, therefore, the choice of model was not limited.

The models used in this part of our study are the most popular ones regarding both their performance on the ImageNet database and their availability. Transfer learning requires a model that has already been trained on a (very) large database and has acquired great knowledge regarding the classification process in various tasks. Moreover, as the target domain (classification of dolomitic marble tiles) is very different compared to the original domain the models were trained on, the choice of the model is not simple nor straightforward. Specifically, the original domain focuses on generalization and object categorization, while the study’s domain is texture classification. For this reason, almost all of the available models were employed and compared their transfer learning performance extensively.

For the transfer learning and fine-tuning process, the following steps were followed:

1. Remove the original output layer
2. Freeze the model’s weights
3. Add a Global Average Pooling 2D layer
4. Add a Dropout layer with a 20% rate
5. Add a Dense layer (output layer) with a softmax activation function for the three quality classes
6. Train only the newly added layers
7. Unfreeze the model’s weights
8. Train the unfrozen weights

**Figure 6:**
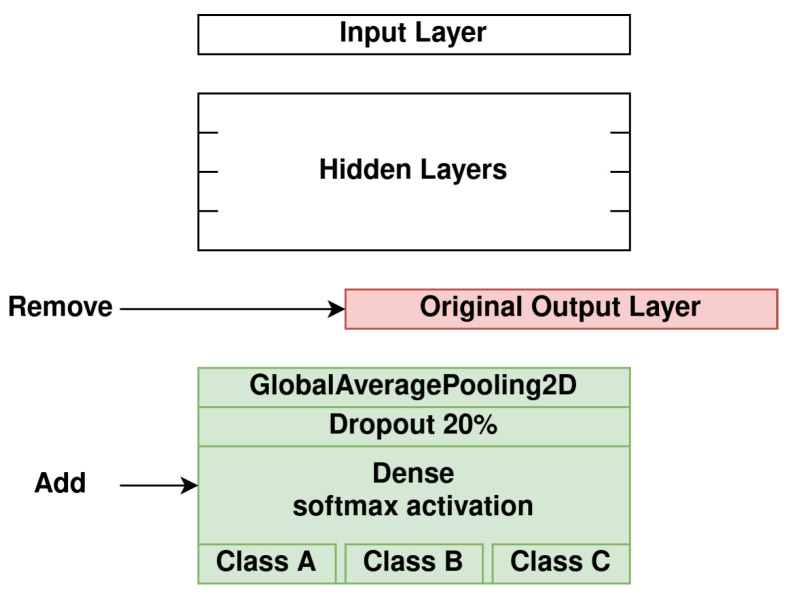
Modification process of the CNNs.

The modification process of the aforementioned CNNs can be seen in Figure 6 (steps 1–5).

For the training and evaluation process of the models, a 10-fold cross-validation technique was employed, splitting the dataset into 90% training set and 10% testing set, with 10% of the training set being used for validation. In each training step (5 and 7), the model was trained for 50 epochs, as their performance started to converge at that point, at the same time trying to prevent any overfitting that may occur. Moreover, during the fine-tuning process, the number of trainable layers that yielded the best results was explored. Specifically, the network’s performance was evaluated by training 25%, 50%, 75% and 100% of the layers (during steps 6 and 7). The fine-tuning process was evaluated by using the F1-score, with accuracy, precision, recall and F1 scores being calculated. For the experiments, the Tensorflow Library was used and Scikit-Learn for performance evaluation. Lastly, the images were resized to 224 × 224 pixels (as per the models’ input requirements), and in each model, they were preprocessed by using each model’s corresponding preprocessing function. It should be mentioned that during the training process, the input images were randomly flipped, both horizontally and vertically, producing augmented training data.

### 3.5. Feature Aggregation

In this step, the methodology proposed by [55] was followed, aggregating the features extracted from the HCDs and the CNN models towards improving the classification accuracy of the constructed marble tiles screening system.

**Figure 7:**
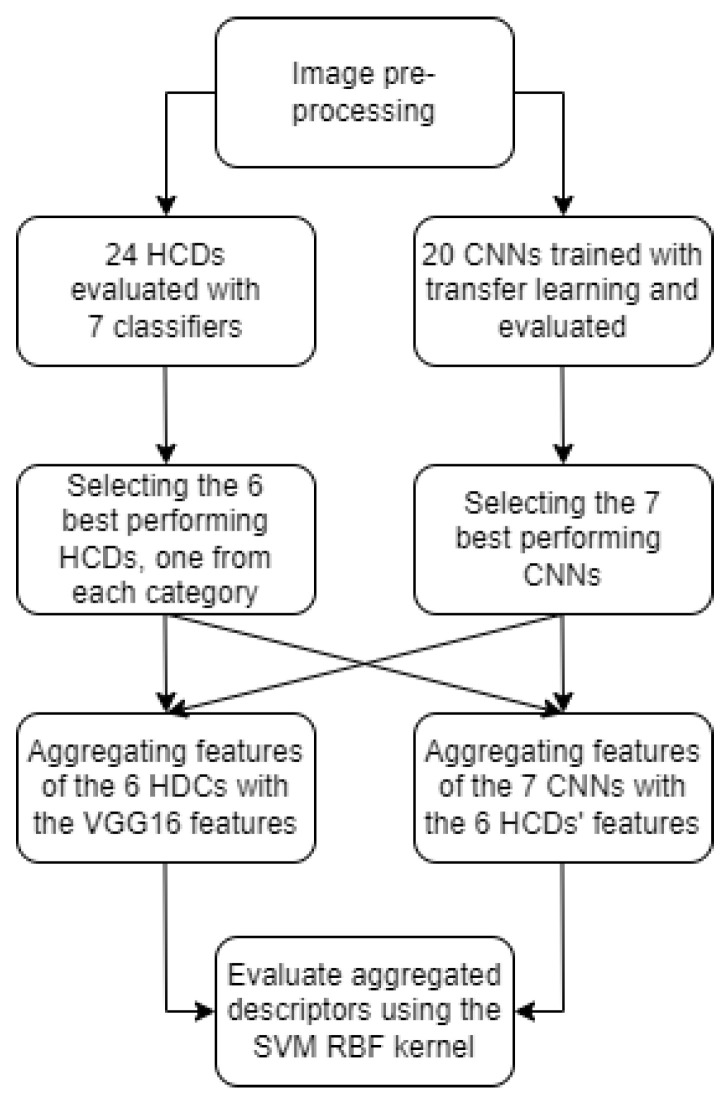
Flowchart of the proposed methodology.

The first step of the feature aggregation process is to combine the feature vector of an HCD and the one of a CNN model. As the two types of features are very different, the choice of which HCD feature vector to aggregate with which CNN feature vector is not simple. Moreover, the evaluation of all the combinations is not feasible either, as the total number of combinations is as high as 480, requiring a lot of computation, while the presentation of its results would be very hard. Therefore, the best HCD in each category and best CNNs of each group were chosen (Figure 7) so that the search space of all combinations is reduced significantly. More specifically, the categories of HCD were the following: LBP, LDP, LMP, LTP, key-point descriptors and the “other” type (as mentioned in Section 3.3 and Table 1). Similarly, from the CNNs, the best model in each group was chosen, with the groups namely being: DenseNet, EfficientNet, ResNet, ResNetV2, VGG, Inception/Xception and NASNet/MobileNet. This way, the complementarity of the two types of features is tested more efficiently by testing combinations with different types of HCDs (ternary, mapped, local, etc.) and all the different types of model architectures in regards to the model’s depth (number of layers) and the number of parameters.

Secondly, for the feature extraction process of the CNN models, an additional layer had to be added before the Global Average Pooling 2D layer, consisting of 1000 units and, as a result, extracting a feature vector of 1000 values. Each model was then trained for 15 epochs (similarly to the transfer learning process, when the model started to converge and before any overfitting happened) on the whole dataset. Then, the output of the Global Average layer was taken as the feature vector for all the images, creating a dataset of CNN-extracted feature vectors for all the available images.

## 4. Results

In this study, 24 HCDs, 20 CNNs and the aggregation between 6 HCDs and 7 CNNs were compared in terms of their performance in classifying dolomitic marble tiles based on their aesthetic value. The experiments were conducted using a desktop computer equipped with a CPU with 12 cores and 24 threads, 32 GB RAM and a GPU with 24GB of VRAM. It should be noted that after finding the best parameters of each model, their performance was evaluated using the leave-one-out cross-validation technique, where in each fold, only one sample is used for testing. Therefore, the cross-validation was performed 441 times, once for each sample. Using the results of this step, the Receiver Operating Characteristic (ROC) curve and the Area Under Curve (AUC) metrics were calculated. The same process was followed during the feature aggregation step, where the model that performed the best in the first case was used. In the case of the experiments where transfer learning was applied, the mean score of the test folds was used as the evaluation score of each model.

### 4.1. Hand-Crafted Features Performance

**Figure 8:**
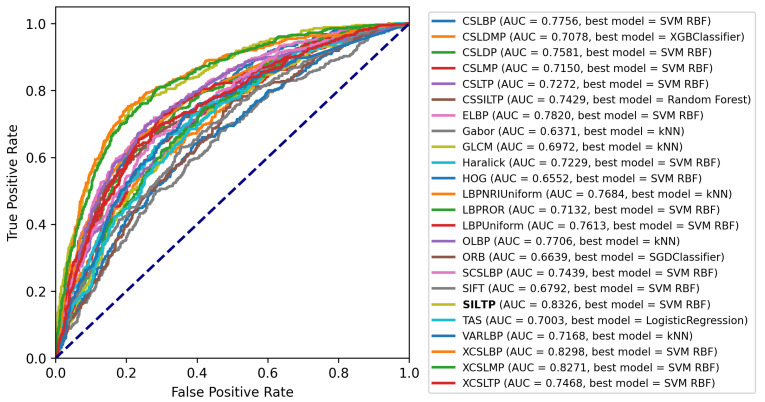
ROC curves and AUC scores of the best performing model for each HCD.

Figure 8 depicts a plot containing a ROC curve and the AUC of the model with the best performance for each HC feature, having a total of 24 lines.

The best performing descriptor was the SILTP, with a 0.8326 AUC score, followed very closely by XCSLBP with a 0.8298 and XCSLMP with a 0.8271 AUC score, all using the SVM RBF classifier. In general, the performance of all the descriptors remains above 0.6371 AUC, with the only descriptors performing above 0.8000 AUC score being the aforementioned ones. The results are presented in more detail in Table 3.

The results show that there is a lot of room for improvement regarding the classification accuracy when extracting local features from the texture images. On average, the HCDs performed poorly in the classification process on the specific dataset, with the best performing descriptor being the SILTP with 66.67% Accuracy, 66.61% Precision, 66.67% Recall, 66.61% and 0.8315 AUC. The XCSLMP descriptor performed about the same regarding the AUC score but better in regards to the rest of the metrics, with 67.12% Accuracy, 67.18% Precision, 67.12% Recall, 67.14% and 0.8314 AUC. The results obtained also highlight that the SVM model’s prediction probabilities (how confident the model is about its prediction) are both low and high; low because the AUC combined with the rest of the performance metrics is not high enough to assume that the model can classify the textures correctly and confidently and high because, when comparing the high AUC with the low F1 metric, it shows that the model is confident about its incorrect predictions.

### 4.2. CNN Learned Features Performance

**Figure 9:**
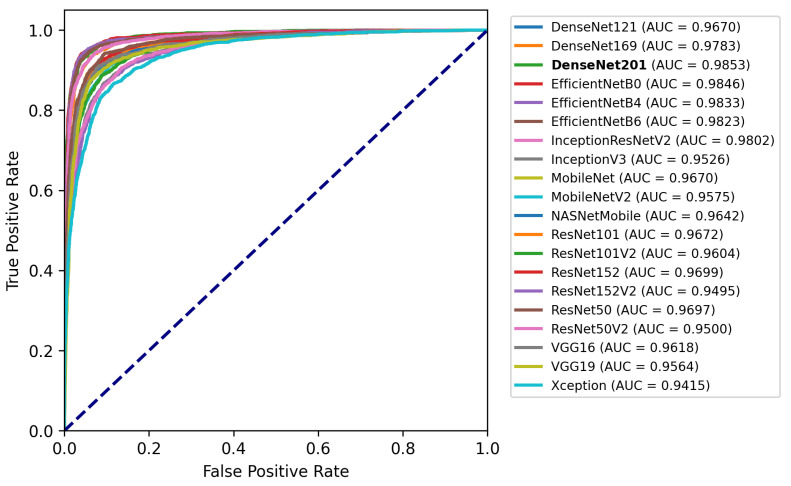
ROC curves and AUC scores of each CNN model.

Figure 9 depicts a plot containing a ROC curve and the AUC for each fine-tuned model.

In this case, the results are much more satisfying, with all the models having an AUC score of above 0.9400 and many of those having above 0.9700. More specifically, the best performing model was the DenseNet201 with a 0.9853 AUC score, followed by EfficientNetB0 with 0.9846, two very different architectures regarding the size of the model. In general, all the EfficientNet models performed above 0.9800, showing that the specific architecture works well in this type of problem. On the other hand, the worst performance was observed by the Xception model, with 0.9415 AUC, followed by ResNet50V2 with 0.9500. The results are presented in more detail in Table 4.

The models, in general, show a high classification rate while also having confident predictions. This is highlighted by the high average scores along with the high AUCs. For example, DenseNet201, which performed the best, has 92.18% Accuracy, 82.49% Precision, 92.18% Recall and 92.05% F1 score.

### 4.3. Aggregated Features Performance

**Figure 10:**
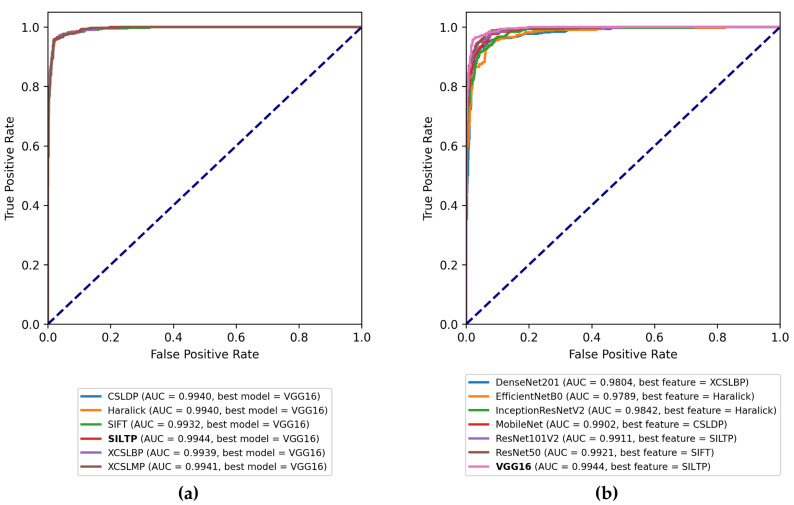
Best ROC AUC scores obtained for (**a**) each HCD along with its best CNN aggregation, (**b**) each CNN along with its best HCD aggregation.

In this step of the experiments, the performance of the SVM RBF model is evaluated, following the same methodology as before, on the aggregated features. Figure 10a shows a plot containing a ROC curve and the AUC score for each combination of features, while Figure 10b shows the best AUC scores of each HCD when aggregated with the features extracted from the CNNs. In the first case, the combinations are different in each case, with the Haralick and SILTP descriptors performing the best in the two aggregated cases. Additionally, the ROC curves in the figure are not distinguishable at all, highlighting two facts: (1) the best performance for each case is quite high, with the lowest being that of 0.9932 AUC, and (2) the comparative performance of the HCDs (when aggregated with the feature vector of generated by the VGG16 network) is negligible, showing that the problem has been solved to a very high degree. In all cases, the best-performing CNN features were that of the VGG16 model, performing above 0.9900 in all cases, with the best combination being with the SILTP feature at 0.9944 AUC. The worst performance was observed by SIFT with a 0.9932 AUC score. The results highlight the assumption that was made in Section 3.5, where the complementarity of the two types of features is not straightforward nor evident.

**Figure A1:**
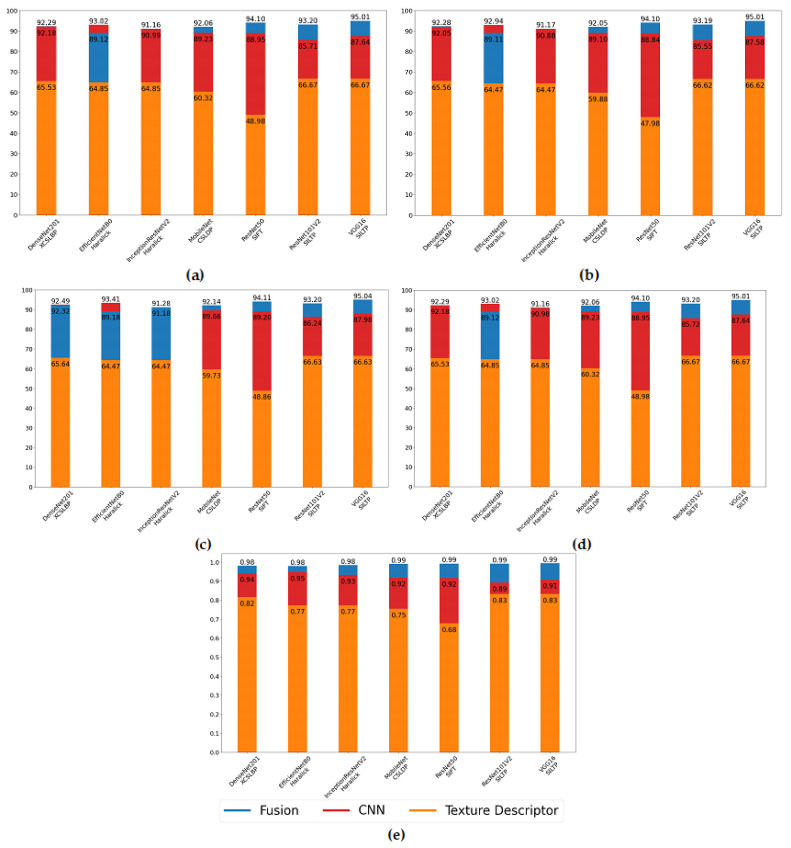
Stacked Bars, (**a**) Accuracy, (**b**) F1, (**c**) Precision, (**d**) Recall, (**e**) AUC score.

In Figure A1, the corresponding stacked bars are presented in a comparative manner. These plots reveal that the applied aggregation strategy explores the complementarity of the hand-crafted and the learned features by providing more efficient aggregated features. The results are presented in more detail in Table 5 and Table 6.

## 5. Discussion

**Figure 11:**
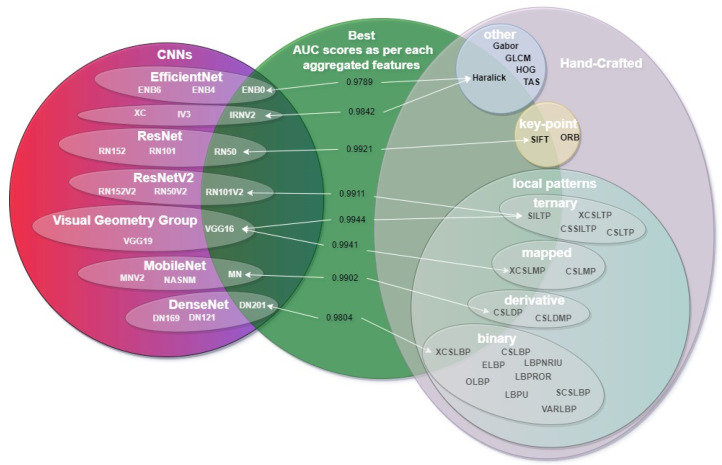
Graphic representation of the experiments and the best AUC scores as per each aggregated descriptor.

The approach proposed in this paper has been formerly used on experimental datasets [55,56]. In this work, real-world natural non-stationary digital images of dolomitic marble tiles, rather than artificially compiled datasets, were used. Furthermore, apart from the LBP HC descriptor used in similar works [55] to aggregate the CNNs, in total 6 HCDs were used in the experiments presented here. The aggregation of the CNNs and HC descriptors produced better AUC scores. In the case of the SILTP HCD, an AUC of 0.8326 was achieved. Furthermore, in the case of the standalone VGG16 CNN, an AUC of 0.9618 was achieved. When these two groups of features were aggregated, the score of the AUC increased to 0.9944. This represents an improvement of 12.92% in the case where only the SILTP HCD was used and 3.27% in the case where only the VGG16 CNN was used. HCDs were also used in the experiments. In Figure 11, the area of the green circle excluded depicts the best AUC scores as per each aggregated feature. It should be noted that the VGG16 CNN was tested with each HC descriptor, and the best-performing HCDs were tested on the seven best-performing CNNs. In order to keep the representation simple, only the best two (XCSLMP and SILTP) out of the six scores are presented in the case of the VGG16. In former works of our team, a similar imbalanced dataset with 986 digital images was used. In this case, the best score for the three class problem was a 65.06% F1-score when using the XCS-LBP texture descriptor with the XGB classifier [8] and the DN201 with an accuracy of 83.24% [57].

Moreover, the results of Figure 10a highlight that smaller (depth-wise) networks complement the features extracted by the HCDs better. In other words, smaller networks extract features that do not “overlap” with the ones that are extracted by the HCDs. As a result, the aggregated feature vector describes almost the entirety of the characteristics of each dolomitic marble quality class. This is highlighted by the fact that all of the HCDs’ best-performing model is the VGG16, which is a very dense network as it has a depth of only 16 layers with a total of 138 million parameters, compared to the larger architectures of DenseNet201 which has 402 layers and (only) 20 million parameters.

On the other hand, each CNN’s best aggregation is different in each case, highlighting the fact that each model and, therefore, architecture and number of parameters extract different features that are complemented by different HCDs in each case. Additionally, almost all of the networks are complemented by different types of HCDs; for example, DenseNet is complemented by an LBP descriptor, while MobileNet by an LDP. Haralick and SILTP descriptors seem to complement more than one network specifically Haralick complements EfficientNetB0 and InceptionResNetV2, while SILTP complements ResNet101V2 and VGG16.

## 6. Conclusions

The results of the experiments indicate that the aggregation of the SILPT HCD and the VGG16 CNN for extracting features performed better compared to the total of 24 HCD and 20 CNNs. This outcome is very important for the development of an efficient visual marble screening machine for the three classes studied here. Each class of the dataset provided by Solakis S.A., and presented in this work, can be subdivided into more than three subclasses. As marketing schemes change, more classes may be necessary to meet customers’ needs. Therefore, our future work involves testing the resulting model on more than the three marble classes used in this work.

Furthermore, better digital images provided by the automatic screening machine constructed by Intermek A.B.E.E. will deliver a better dataset to further tune the model.

## Figures and Tables

**Table 1:** HCD used in the study.

Type of Descriptor		Name
**Key-point Detectors and Descriptors**		Oriented FAST and rotated BRIEF (ORB) Scale Invariant Feature Transform (SIFT)
**Local Pattern Descriptors**	**Local Ternary Patterns (LTP)**	SILTP CSLTP CSSILTP XCSLTP
	**Local Derivative Patterns (LDP)**	Center-Symmetric LDP (CSLDP) Center-Symmetric Local Dritative Mapped Pattern (CSLDMP)
	**Local Mapped Patterns (LMP)**	eXtended Center-Symmetric LMP (XCSLMP) Center-Symmetric LMP (CSLMP)
	**Local Binary Patterns (LBP)**	eXtended Center-Symmetrical LBP (XCSLBP) Center-Symmetric LBP (CSLBP) Elliptical-LBP (ELBP) LBP-NRI Uniform LBP-ROR LBP-Uniform OLBP SCSLBP VARLBP
**Other**		Haralic Gabor GLCM Histogram of Oriented Gradients (HOG) TAS

**Table 2:** CNNs used in the study.

CNN Type	Name
EfficientNet	ENB6 ENB4 ENB0
ResNet	RN152 RN101 RN50
ResNetV2	RN152V2 RN50V2 RN101V2
Visual Geometry Group	VGG16 VGG19
MobileNet	MNV2 NASMN MN
DenseNet	DN169 DN121 DN201
Other	XC IV3 IRNV2

**Table 3:** Validation results obtained for the best performing classifier on average (SVM).

Descriptor	Accuracy (%)	Precision (%)	Recall (%)	F1 (%)	AUC
CSLBP	58.50	58.32	58.50	58.40	0.7743
CSLDMP	56.69	56.69	56.69	56.68	0.7166
CSLDP	60.32	59.73	60.32	59.88	0.7541
CSLMP	53.29	52.97	53.29	52.96	0.7154
CSLTP	55.10	55.95	55.10	55.39	0.7336
CSSILTP	58.05	58.05	58.05	58.05	0.7588
ELBP	62.36	62.20	62.36	62.27	0.7813
Gabor	53.52	54.37	53.52	52.50	0.7098
GLCM	54.42	54.20	54.42	54.08	0.6946
Haralick	64.85	64.47	64.85	64.47	0.7719
HOG	47.39	47.58	47.39	47.08	0.6342
LBPNRIUniform	59.64	59.65	59.64	59.64	0.7939
LBPROR	48.30	48.65	48.30	48.42	0.7056
LBPUniform	57.82	57.37	57.82	57.09	0.7348
OLBP	60.77	60.52	60.77	60.62	0.7741
ORB	40.14	39.09	40.14	38.42	0.6425
SCSLBP	55.78	55.18	55.78	55.17	0.7344
SIFT	47.39	47.52	47.39	47.43	0.6683
**SILTP**	**66.67**	**66.61**	**66.67**	**66.62**	**0.8315**
TAS	51.25	51.05	51.25	51.07	0.7048
VARLBP	51.02	51.38	51.02	51.17	0.6966
XCSLBP	65.53	65.64	65.53	65.56	0.8054
XCSLMP	67.12	67.18	67.12	67.14	0.8314
XCSLTP	55.33	55.80	55.33	55.23	0.7293

**Table 4:** Validation results obtained from the CNN learned features.

Model	Accuracy (%)	Precision (%)	Recall (%)	F1 (%)	AUC
DenseNet121	88.61	89.33	88.60	88.44	0.9670
DenseNet169	90.87	91.40	90.88	90.72	0.9783
**DenseNet201**	**92.18**	**92.49**	**92.18**	**92.05**	**0.9853**
EfficientNetB0	93.02	93.41	93.02	92.94	0.9846
EfficientNetB4	92.97	93.20	92.97	92.89	0.9833
EfficientNetB6	92.35	92.57	92.35	92.25	0.9823
InceptionResNetV2	90.99	91.28	90.98	90.88	0.9802
InceptionV3	84.13	84.65	84.14	83.86	0.9526
MobileNet	89.23	89.66	89.23	89.10	0.9670
MobileNetV2	85.88	86.38	85.89	85.69	0.9575
NASNetMobile	87.30	87.64	87.30	87.14	0.9642
ResNet101	88.15	88.79	88.16	87.95	0.9672
ResNet101V2	85.71	86.24	85.72	85.55	0.9604
ResNet152	88.94	89.45	88.95	88.80	0.9699
ResNet152V2	84.18	84.80	84.17	84.03	0.9495
ResNet50	88.95	89.20	88.95	88.84	0.9697
ResNet50V2	84.01	84.92	84.01	83.74	0.9500
VGG16	87.64	87.98	87.64	87.58	0.9618
VGG19	87.41	87.80	87.41	87.35	0.9564
Xception	82.94	83.29	82.94	82.70	0.9415

**Table 5:** Validation results obtained for each HCD and its best CNN aggregation.

Feature Name	CNN Name	Accuracy (%)	Precision (%)	Recall (%)	F1 (%)	AUC
CSLDP	VGG16	95.46	95.50	95.46	95.47	0.9940
Haralick	VGG16	95.46	95.50	95.46	95.47	0.9940
SIFT	VGG16	95.01	95.03	95.01	95.01	0.9932
**SILTP**	**VGG16**	**95.01**	**95.04**	**95.01**	**95.01**	**0.9944**
XCSLBP	VGG16	95.46	95.50	95.46	95.47	0.9939
XCSLMP	VGG16	95.46	95.48	95.46	95.47	0.9941

**Table 6:** Validation results obtained for each CNN and its best HCD aggregation.

Feature Name	CNN Name	Accuracy (%)	Precision (%)	Recall (%)	F1 (%)	AUC
DenseNet201	XCSLBP	92.29	92.32	92.29	92.28	0.9804
EfficientNetB0	Haralick	89.12	89.18	89.12	89.11	0.9789
InceptionResNetV2	Haralick	91.16	91.18	91.16	91.17	0.9842
MobileNet	CSLDP	92.06	92.14	92.06	92.05	0.9902
ResNet101V2	SILTP	93.20	93.20	93.20	93.19	0.9911
ResNet50	SIFT	94.10	94.11	94.10	94.10	0.9921
**VGG16**	**SILTP**	**95.01**	**95.04**	**95.01**	**95.01**	**0.9944**

## Data Availability

https://github.com/MachineLearningVisionRG/d-dom-dataset (accessed on 6 July 2022).

## Appendix A

The stack bars of Figure A1 depict the performance of each individual CNN model (red) and HCD (orange), as well as their aggregation’s performance (blue).
